# Supplementary material for: Selection and Validation of Reference Genes for qRT-PCR Analysis in the Oil-Rich Tuber Crop Tiger Nut (Cyperus esculentus) Based on Transcriptome Data
Source: Int J Mol Sci. 2021 Mar 4;22(5):2569. doi: 10.3390/ijms22052569 (PMC7961719; doi:10.3390/ijms22052569)
Supplement: Supplementary file 1 [file ijms-22-02569-s001.pdf]

Supplementary Information

Selection and Validation of Reference Genes for qRT-PCR Analysis in the Tuber Oil-Rich Crop Tiger Nut (*Cyperus esculentus*) Based on Transcriptome Data

Xue Bai [1, 2], Tao Chen[1, 2], Yuan Wu[1], Mingyong Tang[1, 3, *], Zeng-Fu Xu[1, 4, *]

1 CAS Key Laboratory of Tropical Plant Resources and Sustainable Use, Xishuangbanna Tropical Botanical Garden, Innovation Academy for Seed Design, Chinese Academy of Sciences, Menglun, Mengla 666303, China; baixue2015@xtbg.ac.cn (X.B.); chentao@xtbg.ac.cn (T.C.); yangyunju@xtbg.ac.cn (Y.W.)

2 College of Life Sciences, University of Chinese Academy of Sciences, Beijing 100049, China

3 Center of Economic Botany, Core Botanical Gardens, Chinese Academy of Sciences, Menglun, Mengla 666303, China

4 State Key Laboratory for Conservation and Utilization of Subtropical Agro-Bioresources, College of Forestry, Guangxi University, Nanning 530004, China

* Correspondence: tangmingyong@xtbg.ac.cn (M.T.); zfxu@gxu.edu.cn (Z.-F.X.)

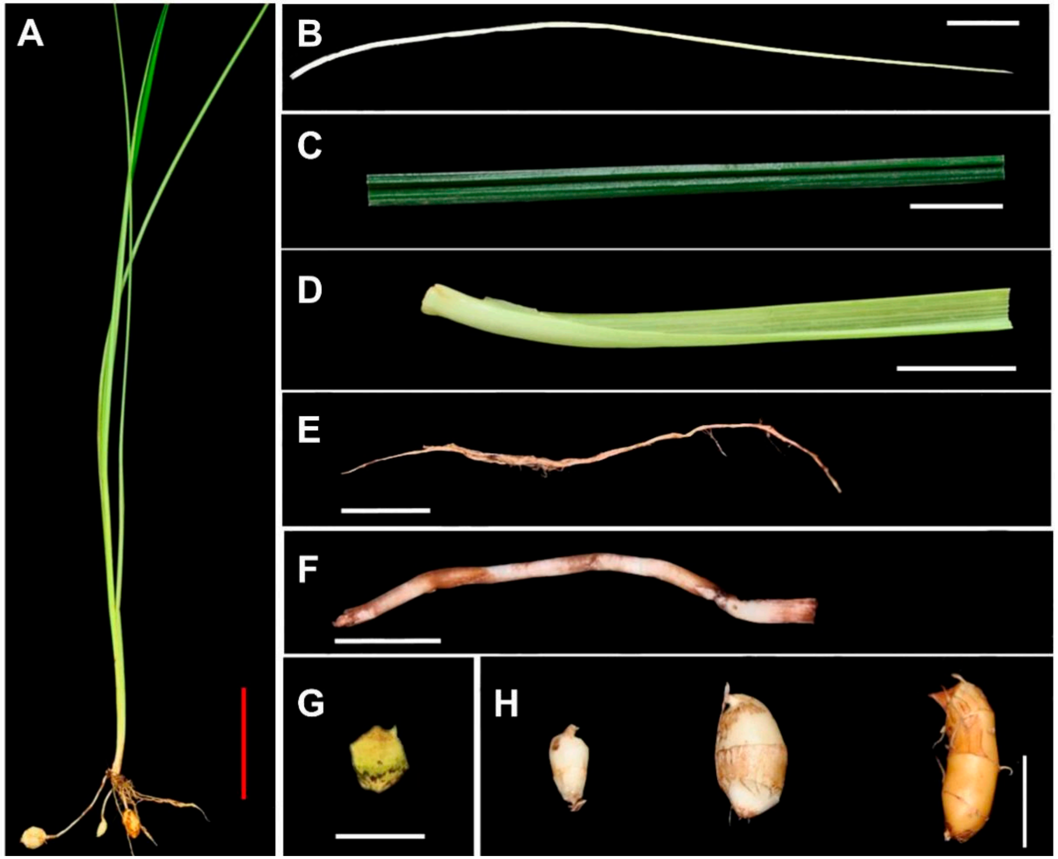

Figure S1 Tissues of C. elesculentus from Yunnan (YN) sampled for RT-qPCR analysis. A-G were harvested at 80 days after sowing (DAS). (A) The whole plant. (B) Young leaf. (C) Mature leaf. (D) Leaf sheath. (E) Root. (F) Rhizome. (G) Stem apex. (H) Three developmental stages of tubers were collected at 40, 80 and 120 days after sowing (DAS), respectively, which are the stages of tuber formation, swelling and maturity in turn. Red bar = 5 cm; white bars = 1 cm.

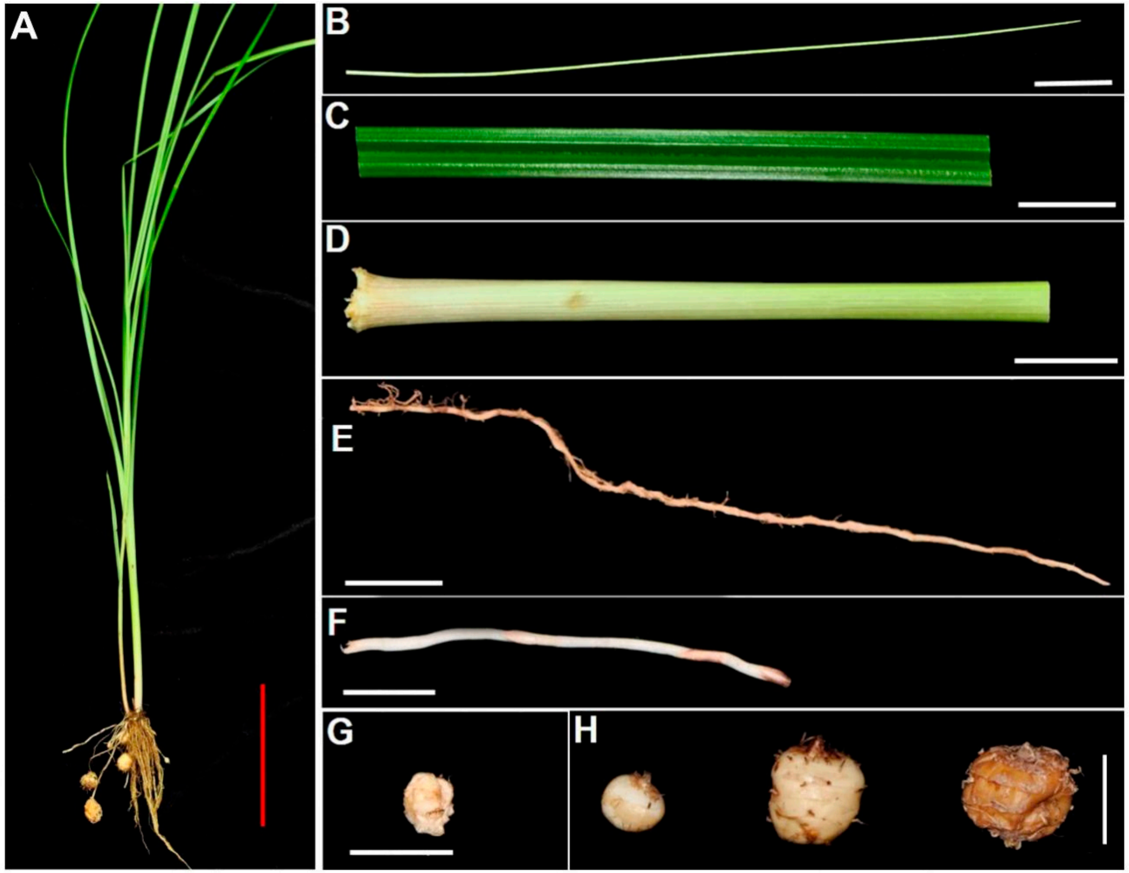

Figure S2 Tissues of C. elesculentus from XinJiang (XJ) sampled for RT-qPCR analysis. A-G were harvested at 80 days after sowing (DAS). (A) The whole plant. (B) Young leaf. (C) Mature leaf. (D) Leaf sheath. (E) Root. (F) Rhizome. (G) Stem apex. (H) Three developmental stages of tubers were collected at 40, 80 and 120 days after sowing (DAS), respectively, which are the stages of tuber formation, swelling and maturity in turn. Red bar = 5 cm; white bars = 1 cm.

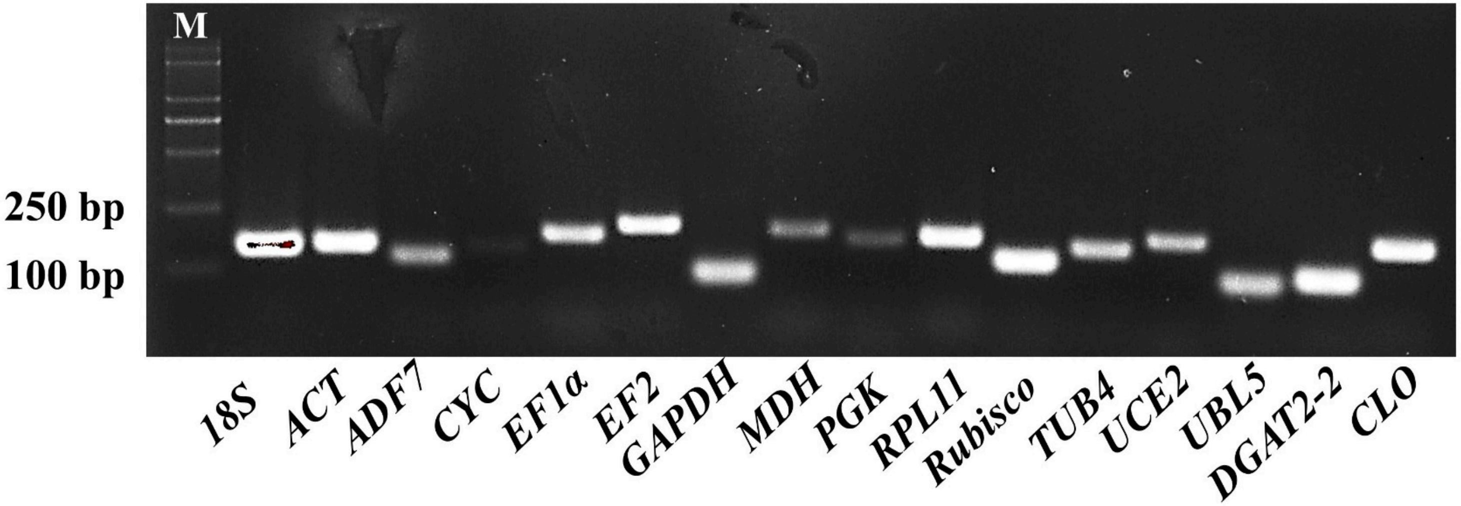

Figure S3 The PCR amplification specificities of 14 candidate reference genes and two target genes (*DGAT2-2* and *CLO*) detected by agarose gel electrophoresis. M, marker (100 and 250 base pair gene rulers were point out).

Table S1 cDNA sequences of 14 reference genes and two genes for validation. The positions of the forward and reverse primers were highlighted in bold and underlined.

18S rRNA (18S ribosomal RNA)

GCCCACATCGCCGCGATCCGAACACTTCACCGGACCATTCAATCGGTAGGAGCGACGGGCGGTGTGTACAAAGGGCAGGGACGTAGTCAACGCGAGCTGATGACTCGCGC
TTACTAGGCATTCCTCGTTGAAGACCAACAATTGCAATGATC**TATCCCCATCACGATGAAATTTCTC**AAGATTACCCGGGCCTTTCGACCAAGGCTATAGACTCGTTGAG
TACATCAGTGTAGCGCGCGTGCGGCCCAGAACATCTAAGGGCATCACAGA**CCTGTTATTGCCTCAAACTTCC**GTGGCCTAAACGGCCATAGTCCCTCTAAGAAGCTAGCT
GCGGAGGGATTCCTCCGCATAGCTAGTTAGCAGGCTGAGGTCTCGTTCGTTAACGGAATTAACCAGACAAATCGCTCCACCAACTAAGAACGGCCATGCACCACCACCCA
TAGAATCAAGAAAGAGCTCTCAGTCTGTCAATCCTTGCTATGTCTGGACCTGGTAAGTTTCCCCGTGTTGAGTCAAATTAAGCCGCAGGCTCCACGCCTGGTGGTGCCCT
TCCGTCAATTCCTTTAAGTTTCAGCCTTGCGACCATACTCCCCCCGGAACCCAAAAACTTTGATTTCTCATAAGGTGCCGGCGGAGTCCTAAAAGCAACATCCGCCGATC
CCTAGTCGGCATCGTTTATGGTTGAGACTAGGACGGTATCTGATCGTCTTCGAGCCCCCAACTTTCGTTCTTGATTAATGAAAACATCCTTGGCAAATGCTTTCGCAGTT
GTTCGTCTTTCATAAATCCAAGAATTTCACCTCTGACTATGAAATACGAATGCCCCCGACTGTCCCTCTTAATCATTACTCCGATCCCGAAGGCCAACACAATAGGACCG
AAATCCTGTG

ACT (actin)

AGGGTTTCGGCGAGAGAGAGATCAGATCACTACTCAGTACTCGCTCTCTCCTCTCCTCCAGTGACACAATTCACAAAGGTTCCTCTTCACAACAAAATTAAAAAATGGCC
GATGCTGAGGATATCCAGCCCCTTGTGTGCGACAATGGTACTGGAATGGTCAAGGCTGGATTTGCTGGTGATGATGCTCCCAGGGCTGTATTCCCCAGTATTGTGGGGCG
TCCTCGCCACACTGGTGTGATGGTTGGCATGGGCCAGAAAGATGCATACGTTGGTGATGAGGCACAGTCAAAGAGAGGTATCTTGACTCTCAAGTATCCGATTGAACATG
GTATAGTCAGCAACTGGGATGACATGGAGAAGATCTGGCATCACACATTCTACAATGAGCTCCGTGTTGCCCCAGAAGAGCACCCTGTGCTCCTCACAGAAGCTCCT**CTC**
AACCCCAAGGCCAACAGAGAGAAGATGACCCAGATCATGTTCGAGACCTTCAATGTACCGGCAATGTATGTTGCCATCCAGGCCGTCCTTTCTCTCTACGCCAGTGGTCG
TACAACTGG**TATTGTTCTTGACTCTGGTGATGG**TGTGAGCCACACAGTCCCAATTTACGAGGGTTATGCCCTTCCTCATGCCATCTTGCGTCTTGACTTGGCTGGCCGTG
ATCTCACTGATGCTTTGATGAAGATCCTGACGGAGAGAGGGTATTCCTTCACCACCACAGCCGAGCGGGAAATTGTGAGAGACATCAAGGAGAAGCTTGCCTATGTAGCT
CTCGACTATGAGCAGGAGCTGGAGACTGCCAAGAGCAGCTCTGCTGTTGAGAAGTCGTATGAGCTTCCTGATGGCCAGGTCATCACCATTGGTGCTGAGAGATTCAGGTG
TCCAGAGGTGCTCTTCCAGCCATCATTGATAGGTATGGAAGCTGGCGGTATCCATGAGACCACCTACAACTCCATCATGAAGTGTGATGTGGATATTAGGAAGGACCTTT
ACGGAAACATTGTGCTCAGTGGTGGGTCTACCATGTTCCCGGGCATTGCTGACCGTATGAGCAAGGAGATCACCGCCCTTGCACCCAGCAGCATGAAGATCAAGGTGGTT
GCCCCGCCTGAGAGGAAGTACTCTGTCTGGATCGGTGGTTCCATTCTCGCCTCCCTCAGCACTTTCCAGCAGGTATGTTGCAATTCTTGTTTTTCTTTTATTTATCTGTT

GCATTGCATGCGTGGTGTTAAATATGTTTTGATTTATGTCCTCTACTACTGCAGATGTGGATCTCAAGGGCAGAGTACGATGAGTCAGGTCCGTCAATTGTCCACCGGAA
GTGCTTCTAGATGAGACGGTGGTGGTGGAGTATTATCGGTTTAATGGTTTGGTGTGTTTATGAATTTTATTTTGAAATTTGTATGGAGAGATGCTGCTACTTATGTACTT
TGGTTGGGTTGATAAGGACCATGTTTTGTTAGATTATGATGATGTGACTTGGTTGCTTTTTTATGTTGCTGTGATTTTTAAAACCGGCTGTAATATTTGTCCCTCCCTAT
GTTACTTGTCATCAAAATTTTATTCTATTGATCTTTAGCTGCAGATTTGC

ADF7 (Actin-depolymerizing factor 7)
AAGCAAAGAAAACCAAGGAAAGGAAGCCAAGCCACCACCAGACACCAGTGTTGTTCTAGTTCTACTTCAGCCCACACCACACGTAACGCAACTCTCAACTCCCCTCTTTC
CCCCTTCCCCTCGCTCGCGCTGCTAGCCTAGCTCCACCGCCTCCTGGATCGCGCCCTTTCCCGAAATGGCGAACGCAGCGTCGGGGATGGCAGTCAATGATGACTGCAAG
CTCAAGTTCCTTGAGCTGAAGGCAAAGAGGACGTACCGGTTCATAGTGTTCAAGATAGATGAGAAGCTTAAGCAGGTCATTGTAGAAAAGGTCGGTGAACCAGCCCAGAG
CTATGAGGACTTCACTGGCAGCCTACCCACCAATGAGTGCCGCTATGCCATCTATGACTATGACTTTGTTACTGAGGAGAACTGCCAGAAGAGCAAGATTTTCTTCATTG
CATGGTCGCCT**GACACCGCAAGGGTAAGG**AGCAAGATGCTTTACGCGAGCTCCAAGGACAGGTTCAAGAGAGAGCTGGACGGCATCCAAGTTGAATTGCAAGCAACTGAT
CCTACTGAGATGGGGCTTGACGTAATTCGAGGCCGTGCAAATTAAATGGATTTACTATTATTATTGCTAAAACTGGGTATCATGGTGAGGTACTTAGTGAATGAATATGT
TTATAGTTCTAAGCATGTCGGTGGTTTGGGCAATTATAATGTTGCGCTCGGGCTTGTTGTTTCTGTGCCCTGGCTAGGTTGTTGTTGGCCTTTTTAAGAATAACTTTCTA
CTTTGGGACTTGCTTTGTTTTGCTGTCTTTGTGAACTGCTTTTATGCGAATCTGTAAGTCTGGCCACCTGGGTCAACATTATGGATTGTGTTAGTTAATATGAAGGATGT
AATGGATTTGACCCTGTTATTTTTTTC

CLO (caleosin)
ATGCAGGGAAACACCGTCAACATGGCTGAAATCAGAGATAGCATCAAGAGTGGAGCAAGAGATGACATGAAGATTATGGCACTTCAGGCGCCGGTGACGGCCGCCCGGCC
GGTCAGGGCTGATCTGGATGATCATATACCGAAGCCATACATGGCGAGAGCTCTGGCGGCGCCCGACATGTACCACCCGGAAGGGAGCAGAGACCACAAGCACTACCACC
TCTCCGTGCTTCAGCAGCACGTCACTTTCTTCGACCTTGATGACA**ACGGCATTGTTTATCCCTGG**GAAACATACGGCGGTGCGCGTAAACTCGGGTTTAATGTGATAATG
TCCTTGCTGATGGCAATAGTAATTAATGTCAGTCTCAGCTACCCGACATTACCGGGCTGGTTCCTGAATCCCTTGTTTCCAATCTATAT**ACACAACATACACAGAGCCAA
ACA**CGGCAGCGACTCTGGGTCCTACGACACCGAGGGAAGATACATGCCGGTTTATTTCGAGAACATCTTCAGCAAATATGCACGTACATCTCCCGACAAGCTCTCGTTTA
GAGAGTTGTGGTCCATGACCGAGGGAAACAGGCAGGCTTACGATCTCTTCGGCTGGTTTGCGGCGAAGTTCGAGTGGATCGCATTGTACTTATTGGCAAGGGACGAGGAC
GGCTACCTGTCGAGGGAGGCGATAAGGCGTTGCTTCGACGGGAGCTTGTTCGAGTACTGCGCGAAGCAGAGGGAAGCCAACATGAAGACTTACTAG

CYC (cyclophilin)

TCTTTCATTCAACCAAAAACCCTAGCGACCGCGATCGAGCAAACCATAAAACGGCGATAGAGCTGCTGCTCAGCTAAAACCCTAGTCTCAGTCTCCCTTCCCTTCTTCAT
CTTCCTACGCTTCACGCGGGCAATCAGGAGAGGGCAATCCTTTCAACCATGGCAAATCCCAAAGTCTTCTTTGACATTGCGATCGGCAAGGCCAGGGCCGGACGCATCGT
CATGGAGCTATTCTCGGACGTTGTTCCAAAGACCGCTGAGAATTTCCGCGCCCTCTGCACC**GGTGAAAAGGGTATCGGTC**AAGCCGGCAAGGCATTGCATTACAAAGGCT
CAACCTTCCACCGCATAATCCCCAATTTCATGTGCCAGGGGGGCGACTTCACCCGTGGGAACGGTACCGGCG**GCGAGTCCATCTACGGAAA**CAAATTTGACGATGAGAAC
TTCAAGTTGAAGCACACCGGTCCCGGCGTGTTGTCGATGGCGAACGCCGGTCGCAACACGAACGGCTCGCAGTTCTTCATCTGCACCGCCAAGACTCCGTGGTTGGACGG
GAAGCACGTGGTGTTCGGGAAGGTGGTGGAGGGTTATGGGGTTGTGGAGGCCATGGAGCGGGTCGGCTCTCCAAGTGGATCCACATCCGACAATGTCGTCATTGAGGATT
GTGGTGAGATCAAACAATGATATGTCGCTTTGTAATCTCGGAATCTGGTATGATCAGTGATGCAAGCATACACAGATTTAGTGGCCAGTAACTATAGTTTTTTAGAGAAT
GGTGCTAGGCTAGATTTTTGGTTATAGAGGGGTTAAAACTTGTAATGAAACTTTTGTTGTTGCTTAAAAACAAGATATGAAATTTTCATCTATCTTATCCTTTGTCTATA
TTTCTTGGACAGGTGATTTCTGTTTCAACTTCAGTTCATGGCACATTTGTGTTTCAACTTCAGTTCGTA

DGAT2-2 (diacylglycerol o-acyltransferase 2-2)

GTCTCAACAATGGGAAACAAGGAAACAATGGAAACACCCAAAACTAATACAAGGCCACCACTGCTGAAGAGCATAGTGGCCCTCTTCCTATGGATGGGTATCATACAGCT
CAATGTTGTGCTACTGGTAACGGCCCTCTTCGTCCCGCTTCGCATCGGCACCATGATTATTGGGTTTCTTGTTTTGATGAGGTTGTTGCCTGTCAATCCTAAGAGCAAAT
TTGGAAGCAAAGTTGCAAAGTTTATAGACAAAAATGCATATGGATATTTCCCAATTACAGTTCATTTGGAGGATGAAAAGGCTTTTGATCCCAACCAAGCTTATGTATTT
GGGTATGAGCCGCACACAATATATGCTCTTGGAGCATGGGCACTAACAGACCGCAGCAGCTTGGCACCTGTACCAAAGATCAAGTTTACTGCTGCCAGCATTGCATTCAA
CATTCCAATTCTGAGGCATATTTGGACATGGCTGGGTCTTGTTCCCGTAACCAGAAAGAGTTTCATCAAGCAATTGTCAGCCGGAAACAGCTGCATTGTTGTAC**CAGGTG**
GTGTTCAAGAGATGCTTCATTTGGAGCAGGATTCAGAGGTTGCTTTTCTGAAATCAAGGAAGGGATTTGTAAAATTGGCAATTGAGATGGGCTCCCC**ACTTGTCCCTGTT**
TTCTCCTTTGGTCAGAGCCAAGCATACAAATGGTGGAGACCTCAAGGAAAAATATTTCACAAAATTTATAAGATAGTCAAGCAGCCAATAGTTTTATCCTGGGGAAGATT
TGGGTCACCAATTCCATTTCGAGTACCAATGCACATTGTGATAGGTAAACCAATTCAGCTCAAGAAGAATGCTCAGCCTACTAATGATGAGGTGAATGAAGTGCATGCAC
AATTTGTAGCGGCAATGCAGGAGCTATTTGAGAAACACAAAGCTCAGT

EF1α (elongation factor 1-alpha)

CATCCATCCCTTGGGTTTTTGATTATTCTCTCCATCCATCTGTAGAAGTGTAGTAGGCTATTAATTGTGTACTTCTTGGCACACAAGCAGCCTAATCTTCCTGAGCTTAA
TTTATAAGTCAGCGCAGCAGCCCCCTTTCCCTCACACAACCCAGTCTCCCACTCTCTGTTTTAGCTTTCGCTGCTCGCACCATGGGTAAGGAGAAGGTTCACATCAACAT
TGTGGTCATTGGCCATGTCGACTCTGGCAAATCCACCACCACTGGTCACCTTATCTACAAGCTTGGCGGCATCGACAAGCGTGTCATTGAGCGGTTTGAGAAGGAGGCTG
CTGAGATGAACAAGAGGTCATTCAAGTACGCGTGGGTGCTCGACAAGCTCAAGGCCGAGCGCGAGCGTGGCATCACCATCGACATTGCCCTCTGGAAGTTCGAGACTACC

AAGTACTACTGCACAGTTATTGATGCCCCGGGTCACAGGGATTTCATCAAGAACATGATTACTGGTACATCTCAGGCTGACTGTGCCGTGCTTATTATTGACTCGACCTC
TGGTGGTTTTGAGGCTGGTATTTCCAAGGATGGTCAGACCCGTGAGCATGCGCTTCTGGCCTTTACCCTTGGCGTCAGGCAGATGATCTGCTGCTGTAACAAGATGGATG
CCACCACTCCCAAGTACTCGAAGGCAAGATACGATGAAATTGTGAAGGAAGTCTCGTCTTACCTGAAGAAGATCGGGTACAACCCTGACAAAATCCCATTTGTTCCCATC
TCCGGGTTTGAGGGAGACAACATGATCGAGCGCTCCACCAACCTCGACTGGTACAAGGGTCCCACCCTTCTTGAGGCTCTTGATCAGATCAATGAGCCCAAACGCCCCTC
TGACAAGCCCCTTCGTCTCCCGCTTCAGGATGTCTATAAGATTGGTGGCATTGGAACTGTCCCCGTTGGTCGTGTTGAGACTGGCATCATTAAGC**CTGGTATGCTTGTGA**
CATTTGGTCCCACCGGTCTGACTACTGAAGTGAAGTCTGTGGAGATGCACCACGAGGCTCTCCAAGAGGCACTCCCTGGTGACAACGTCGGCTTCAACGTGAAGAATGTC
GCTGTCAAGGATCTGAAGCGTGGTTACGTGG**CCTCCAACTCCAAGGACGA**CCCTGCCAGAGAGGCTGGAAGCTTCACCTCTCAGGTCATCATCATGAACCACCCTGGCCA
GATTGGCAACGGTTATGCTCCTGTGCTCGATTGCCACACCTCGCACATTGCTGTCAAGTTCTCTGAGATCTTGACCAAGATAGACCGTAGGTCTGGTAAGGAGCTCGAGA
AGGAGCCCAAGTTCTTGAAAAACGGCGATGCTGGGTTTGTTAAGATGATTCCCACAAAGCCCATGGTTGTGGAGACATTCTCCGCGTATCCACCTCTCGGTAGGTTCGCT
GTCAGAGACATGAGGCAGACTGTGGCTGTTGGCGTCATCAAGGCTGTTGAAAAGAAGGATCCTACTGGCGCCAAGGTCACCAAGGCTGCTCAGAAGAAGAAATGAATAAA
CAAGATGGTTCTCTCAGACTGTTCTCGAGAGATCGATTATAGCAGTTATCAGTGCAATAAATCCGTTGATTTGGTATCTGGTATCTGGTTGCAGAACTGGGTTCTCGATC
GACGGTGGTCCTGTTTATAATTTTAGTATGATTATTATTGTGTATTCCGTATATCGAGTGATTTTTTGTGAATGGTCAGTGTGGGACTGCTTATAGTTTGGTTGTGATTG
CTACCATATGGTATATACTTGTCAGTCACTCTGTTATTGGGATGAATCCCCAGTACATATATATTCAGATAAATATATTATATATAGAT

EF2 (elongation factor 2)

AGAGACCTCTCACTTCTCTCATTCTCTCCTTCCTTTCCATTCCTCTCTTGGGCGCCGCCGCCGAGAGATTTTCCTTTCTCACCCTCCCGATCTACGCCCTCCCGACCGAT
CGCCTCCTTCTCATCTAGGATATTTTTTGAGGTTTGTGTGAACTAAGAGATCAGTCAAGATGGTGAAGTTCACAGCAGAGGAGCTCCGGGCCATTATGGACCTGAAGCAT
AACATCAGGAACATGTCCGTCATTGCCCATGTCGATCATGGAAAATCAACTCTTACCGATTCGCTTGTGGCTGCTGCTGGTATCATTGCTCAGGAAGTTGCTGGTGATGT
CCGTATGACTGATACTCGTGCTGATGAGGCTGAGCGTGGTATTACCATTAAATCTACTGGTATCTCACTCTACTATGAGATGACTGATGAAGCTCTCAAGAGCTACAAGG
GAGAGCGCTCAGGCAATTCATACCTTATCAACCTTATCGACTCACCTGGGCACGTTGACTTCTCCTCTGAAGTCACTGCCGCATTGCGTATCACTGATGGTGCCTTGGTT
GTTGTTGACTGTATCGAGGGTGTCTGTGTCCAGACTGAAACTGTGCTCCGTCAGGCTCTTGGTGAGAGGATCAGGCCTGTCTTGACTGTCAACAAGATGGACCGTTGCTT
CCTTGAGCTTCAGGTTGATGGAGAGGAGGCATACCAGACCTTCCAGCGTGTGATTGAGAATGCCAATGTCATCATGGCCACATATGAGGACCCACTTTTGGGTGATGTGC
AAGTGTACCCAGAGAAAGGTACAGTTGCGTTCTCTGCTGGTCTTCATGGCTGGGCTTTCACACTGACCAACTTTGCCAAGATGTATGCCGCAAAGTTTGGTGTTGATGAG
TCTAAGATGATGGAGAGGCTTTGGGGTGAGAACTACTTCGACCCTGCCACCAAGAAGTGGACCTCCAAGAGCACTGGGTCCCCCACTTGCAAGCGTGGATTTGTTCAGTT
CTGCTACGAGCCAATCAAGCAAATCATTGCCACTTGTATGGCTGACCAGAAGGATAAGTTGTGGCCCATGCTTCAAAAGCTTGGTGTGACCATGAAGTCTGATGAGAAGG
ATCTTGTTGGCAAGGCCTTGATGAAGCGTGTCATGCAAACCTGGCTCCCTGCCAGCAATGCTCTTCTAGAGATGATGATATTCCACCTCCCGTCTCCTAGTACTGCTCAG

AAGTACCGTGTTGAGAATTTGTACGAGGGTCCCCTTGATGACATTTATGCCACTGCAATCAGGAACTGTGACCCAGAAGGTCCACTCATGCTCTATGTGTCGAAAATGAT
TCCAGCCTCTGATAAGGGTCGGTTCTATGCCTTTGGTCGTGTGTTTGCTGGAAGAGTTGCTACTGGTATGAAGGTCAGAATCATGGGTCCCAACTATGTTCCTGGTCAGA
AGAAAGATCTGTATGTGAAGAGTGTTCAGAGAACTGTTATCTGGATGGGTAAGAGACAAGAGTCTGTTGAGGATGTGCCATGTGGTAACACTGTTGCCATGGTTGGTCTG
GATCAATTTATCACCAAGAATGCCACTCTAACTAATGAGAAGGAGGTTGATGCCCACCCAATCCGTGCCATGAAGTTTTCAGTGTCCCCTGTCGTGCGTGTTGCTGTCCA
GTGCAAGGTTGCATCTGACTTGCCCAAGCTGGTTGAAGGACTGAAGCGTCTCGCAAAGTCTGACCCTATGGTTGTCTGTAGTATTGAAGAATCTGGTGAGCACATCATTG
CTGGTGCTGGTGAGCTTCACCTTGAGATCTGTTTGAAGGATCTCCAGGAGGATTTCATGGGTGGTGCTGAGATTGTTGTCTCACCACCTGTTG**TGTCCTTCCGTGAGACC**
GTACTTGAGAAGTCGTGCCGCACTGTCATGAGCAAGTCCCCCAACAAGCACAACCGTCTTTACATGGAGGCTCGCCCACTGGAGGAGGGTCTTCCTGAGGCCATTGATGA
GGGCCGCATTGGCCCACGTGATGACCCCAAGGTACGCTCCAAGATCCTCTCCGAGG**AGTTCGGATGGGACAAGGA**TCTTGCCAAGAAGATCTGGTGCTTCGGACCTGAGA
CAACTGGCCCCAACATGGTGGTTGATATGTGTAAGGGAGTTCAGTACTTGAATGAAATCAAGGATTCTGTTGTTGCTGGTTTCCAGTGGGCCTCAAAGGAAGGTGCCTTG
GCTGAGGAGAACATGCGTGGTATCTGTTTTGAGGTATGTGATGTTGTATTGCACGCTGATGCTATCCACCGTGGTGGTGGCCAGGTCATCCCAACTGCCAGGCGTGTGAT
CTATGCTTCCCAGCTGACAGCCAAGCCCAGGCTGCTTGAGCCAGTTTACTTGGTTGAGATCCAGGCCCCTGAGGGTGCCCTTGGTGGTATCTATGGTGTGCTGAATCAGA
AGAGAGGACACGTGTTCGAGGAAATGCAGAGGCCTGGTACCCCTCTGTACAACATCAAGGCATACCTACCGGTTATTGAGTCCTTTGGTTTCTCCAGTACATTGCGTGCT
GCCACCTCTGGTCAGGCTTTCCCACAATGTGTCTTTGATCACTGGGATATGATGTCGGCTGATCCACTTGAGGCTGGTTCCCAGGCTGCCACCATCGTCCAAGAGATCCG
TAAGAGGAAGGGATTGAAGGAGCAGATGACCCCACTATCAGACTTCGAGGACAAGATCTAAATGTGGTAACCTATCTTATGGTCTGTGCCTGTGTGCACATAGTTTTGCT
TCTTGTTGCTGTGTTATTTTTGGACTGTATCAGTTGTTGTGATGTCATTGGAACAATCTCTGTTGTTTTTAAGTGTAGTTCGTGCAGTGTCTTTTGAACCGTGGTGACAT
TTTGCTGAAAGTATGTGAACGGTTTTATATTTTTACTAGATTAGTATGTTGAGTCTGTAGCTTTACAATTTGGACAATGACTGAGAATTGTGTTTTTGGTTTTGATT

GAPDH (glyceraldehyde-3-phosphate dehydrogenase)
ATCGTCTGTGATTTCTCTTCTTCTTTCTCGAATCGCTTAATCCCTGTTATACCCTCGACCTCCCTCGTCCTCCTCTTCGTATTACCATGGGCAAGATCAAGATCGGAATC
AATGGATTCGGAAGGATCGGGCGTCTGGTCGCCAGAGTTGCGCTCCAGAGTGAGGACATCGAGCTCGTCGCCGTCAACGACCCCTTCATCACCACTGACTACATGACCTA
CATGTTCAAGTATGATACCGTACACGGCCAATGGAAGCACCATGATATCTCTGTCAAGGACAGCAAGACCCTTCTCTTCGGTGAGAAGCCCGTAACTGTCTTTGGTTGCA
GGAACCCTGAGGAGATTCCATGGGGTGAGGTCGGTGCTGAGTACGTTGTTGAGTCCACCGGTGTCTTCACCGACAAGGACAAGGCTGCGGCTCACTTGAAGGGTGGTGCC
AAGAAGGTCGTCATCTCAGCTCCCAGCAAGGATGCCCCTATGTTTGTGGTTGGTGTCAACGAGAAGGAGTACAAGTCTGACATTGACATTGTCTCCAACGCTAGCTGCAC
CACCAACTGTCTTGCTCCCCTTGCCAAGGTCATTAATGACAAGTTTGGCATTGTTGAGGGTTTGATGACCACTGTTCACTCTATCACTGCTACCCAGAAAACTGTTGATG
GTCCCTCTGCCAAGGACTGGAGAGGTGGAAGGGCTGCTAGCTTCAACATC**ATTCCCAGCAGCACTGGTG**CTGCCAAGGCTGTTGGAAAGGTGCTTCCAGCTTTGAACGGC

AAGTTGACTGGTAT**GGCTTTCCGTGTGCCAACT**GTTGATGTCTCCGTTGTTGATCTCACTGTCAGACTTGAGAAGGCTGCTTCCTACGATGAAGTCAAGGCTGCCATCAA
GGCCGAGTCTGAGGGCAACCTCAAGGGCATCTTGGGCTACACCGAGGAGGACCTCGTATCAACTGACTTCGTTGGTGACAGCAGGTCTAGCATCTTTGATGCCAAGGCTG
GTATTGCTCTCAACAAAAACTTTCTCAAGGTTGTGTCATGGTACGACAACGAGTGGGGCTACAGCAACCGTGTTGTTGACCTCATCCGCCACATGTCCAAGGTTTAATTT
GTGTTGATATAGGATGCCGTTGTGATCTTATGCCAGTAGGAGGGATCTTTGTGTTTTTCTTTTTCTTTCTCTCAGTATGTTGAATAAAAGTTTTGCGCATCGTACTTTTG
AGAAAACCCAGCTATGCTGAGTTTGTTTTGTATGTTGGCGGGGTAATGACGCTTGTTTTTGCTACTATTGGGATCTGAACTTTTGGTTAATTAAAAAATCTAATTTGGAT
GTGTGAACTGTGAAGTGTGTTT

MDH (malate dehydrogenase)

ATGCAATCCAGCGCTGAACGTGTTGCCCGGATAGCCGGCCACCTCCACCCTCCCACCTCTCAGATGGAGGAGATATCGCTGCTGAGGCAGTCCATCTGCAGGGCGAAAGG
TGGTTCGTCGGGCTTCAAAGTTGCCATCCTTGGAGCGGCTGGTGGTATCGGGCAACCTCTGGCAATGCTCATGAAGATGAACCCTCTTGTGTCGGTTCTTCATCTCTATG
ACGTTGTGAATACCCCTGGTGTCACGGCAGATCTGAGCCACATGAACACCGGTGCTGTGGTACGGGGTTTCATGGGTCAGGCCCAGCTAGACAATGCACTAACCGGGATG
GACCTAGTAGTAATCCCAGCCGGTGTACCTCGTAAACCAGGCATGACAAGAGATGACCTATTCAACATCAACGCGGGGATTGTGCGCACACTCTGTGAGGGGATCACCAG
GTGTTGTCCTAATGCCATTGTCAATGTGATAAGCAACCCTGTCAACTCAACCGTACCTATAGCCGCAGAGGTCTTTAAGAAGGCTGGCACATATGATCCAAGAAAGCTGT
TAGGTGTGACGACACTGGACGTTGTCCGAGCTAATACTTTTGTGGCAGAAGTATTGGGACTTGACCCAAGGGATGTAAATGTTCCAGTAGTTGGTGGCCATGCTGGTGTG
ACAATTCTGCCACTCCTTTCCCAGGTGAACCCTCCTTCGAGCTTCACACCTGAAGAAATCACTTATTTAACTAACCGCATACAGAATGGGGGAACTGAAGTAGTTGAGGC
AAAAGCTGGTACAGGTTCAGCGACATTGTCA**ATGGCTTATGCTGCTGCTC**GTTTTGCTGATGCATGCTTGCGTGGATTGCGTGGAGATGCTGGTGTTGTGGAGTGCTCCT
TTGTTGCTTCTCAGGTTACCGAGCTCCCTTTCTTTGCCTCAAAAGTCCGGCTAGGTC**GGGGAGGTGTGGAGGAAAT**ATTGCCCCTGGGTCCATTAAATGAATTTGAGAGG
TCTGGATTGGAGAAGGCAAAGAAGGAACTGGCAGAGAGTATACAGAAGGGAGTTTCTTTCATTAAGAGCTGA

PGK (phosphoglycerate kinase)

AAATGAACTTTCTTTTATATCTGTAAGCTTACATAATAATAAAGTCAAACAGAGATGCCAACTATGCACTCAATTCCAATAACAAAAAATACTTGGCCGAAATGGTCCAA
GGTGAACATCAGAGACCTTATTTCTGACAGGACAACAATAGCATCATCAAATTGCTAAAGACAGAATGCAATTTCTATTTGCCACTCATAC**AGAAACCAAGGCTTCGTCA**
AGTGCATTTACACCGGGGAGCTCTTTCCCTTCTAATAATTCCAAGGCAGCACCACCGCCAGTAGAGATGTGGCTCATCACACTAGCCACTCCCACCTTCTCTACAGCAGC
AACCGAATCACCACCTCC**AATGATGGTTGTGACTCCCTT**TCCACTTAGATCAGCCAATTTCTTGGCAATCGCCTCAGTTCCGGTAGCAAACTTGTCGAACTCGAAAACTC
CCATGGGTCCATTCCAGATAACCGTCTTGGTAGTTTCCAAAGCCTCACTGAATGTTTTGATAGAATCTGGGCCAATATCTAGACCCATCCAGCCATCAGGATAGCAGAA

GCTGGGACTATCTTGCTGTCTGCATCAGGTGCAAATTTGTCAGCAACTACAACATCAGTTGGAAGCAAAAGAGAGACCCCCTTCTCCTTTGCCTTTGCCATAAGAGAACT
AGCAAGGTCAAGCTTGTCAGCTTCCACAAGCGAGGAGCCAACTGATAACCCCTGTGCCTTATAGAAAGTGAAGATCATACCGCCTCCCAAAATAAGGACGTCACACTTCT
CCAGCAGGGATTCAATAACTCCGATCTTGGATGAGACCTTCGAGCCTCCCACAATAGCCGCAAATGGTCTTGTAGGACTAGAGACAGCTCCGACTAGATAGTCAAGTTCC
TTTTGTAAAAGGAAACCAGCAACCGATGGCTTGAGATACTTGGTGACACCCTCAGTGGA

RPL11 (ribosomal protein L11)
CCGCCATTTCCTCCCTCCCTCCCTTCCTTCAGAGTCGCCGCTGCGGAGCTGCCCCGACACACCTCGAACATCCCTTCAATGGCGTCGGAGAAGAAGCTGTCGAACCCCAT
GAGGGAGATCAAGGTCCAGAAGCTCGTCCTCAACATCTCCGTCGGAGAGAGCGGTGACCGTCTCACCAGGGCTGCGAAGGTGTTGGAGCAATTGAGTGGACAGACCCCCG
TCTTCTCCAAGGCTAGGTACACTGTGCGTTCCTTTGGTATCAGGCGTAATGAGAAGATTGCATGCTACGTGACAGTTAGAGGTGAGAAGGCCATGCAACTGTTGGAGAGT
GGGTTGAAGGTGAAGGAATACGAGTTGCTCAGGAGGAACTTCAGTGAAA**CTGGATGCTTTGGATTCGG**TATCCAAGAGCACATTGATCTTGGCATCAAGTACGATCCGTC
AACTGGTATCTACGGTATGGATTTCTACGTGGTTTTGGAGCGTGCCGGCTACCGTGTGGCACGTAGACGTAGGTGCAAGGCACGTGTTGGTA**TCCAGCACAGAGTTACCA
AG**GAGGATGCCATGAAGTGGTTCCAGGTCAAGTATGAGGGTGTCATCCTTAACAAGTCCCAGGCGAACACCTCTTAA

Rubisco (ribulose bisphosphate carboxylase/oxygenase)
ATGTCACCACAAACAGAGACTAAAGCTAATGTTGGGTTTAAAGCAGGGGTTAAAGATTACAAACTTACTTATTATACTCCTGAGTACGAAACCAAAGATACTGATATCTT
GGCAGCGTTCCGAGTAACTCCTCAACCTGGAGTCCCTCCTGAAGAAGCAGGAGCTGCAGTAGCGGCGGAATCTTCTACTGGTACATGGACAACTGTTTGGACTGATGGAC
TTACCAGTCTTGATCGTTACAAAGGGCGATGCTATCATATCGAACCTGTTGCTGGAGAAGAAAATCAATATATTGCCTATATAGCTTATCCTTTAGACCTTTTCGAAGAA
GGTTCTGTTACTAACATGTTTACTTCTATTGTAGGTAATGTATTTGGTTTCAAAGCCTTACGAGCTCTACGCTTGGAAGACTTACGAATTCCTCCTGCTTATTCAAAAAC
TTTCCAAGGTCCACCTCACGGTATCCAAGCTGAAAGAGATAAGTTGAACAAGTATGGTCGTCCTCTATTGGGATGTACTATTAAACCAAAATTGGGATTATCCGCAAAGA
ATTACGGTAGAGCATGTTATGA**ATGTCTACGTGGTGGACTTGAT**TTTACCAAAGATGATGAAAACGTAAACTCACAACCATTTATGCGTTGGAGAGATCGTTTCTTGTTC
TGTGCCGAAGCAATTT**ATAAAGCACAAGCCGAAACA**GGTGAAATCAAAGGGCACTACTTGAATGCTACTGCAGGTACATCTGAAGAAATGATCAAAAGAGCAGTATTTGC
TAGAGAATTAGGAGTTCCTATCATCATGCATGACTACATAACTGGGGGATTCACTGCAAATACTAGTTTGTCTTTTTATTGCCGTGATAATGGTCTACTTCTGCACATCC
ACCGCGCAATGCATGCAGTTATTGATAGACAGAAAAACCATGGTATTCATTTCCGTGTACTAGCTAAAGCATTACGTATGTCTGGTGGAGATCATATTCACTCTGGTACA
GTAGTAGGTAAACTGGAAGGTGAGCGTGAGATGACTTTAGGTTTTGTTGATTTACTACGTGATGATTATATTGAAAAAGATCGTAGCCGTGGTATCTTTTTCACTCAAGA
TTGGGTCTCTATGCCTGGTGTTATACCTGTGGCTTCAGGGGGTATCCATGTTTGGCATATGCCTGCTTTGACCGAAATCTTTGGAGATGATTCTGTACTTCAATTTGGTG
GCGGAACCTTAGGACACCCTTGGGGAAATGCACCTGGTGCAGTAGCTAACAGGGTGGCTTTAGAAGCGTGCGTACAAGCTCGTAATGAAGGACGTGATCTTGCTCGTGAA

GGTAATGAAATTATTCGCGCAGCAGCTAAATGGAGTCCAGAATTAGCCGCTGCTTGTGAAGTATGGAAAGCAATCAAATTTGACTTCGATCCGGTAGATAAACTAGATAA
AGCGAAATAG

TUB4 (tubulin beta-4)

ATGAGAGAGATCCTCCATATCCAAGGTGGTCAATGTGGTAACCAGATCGGTGCCAAGTTTTGGGAAGTCGTCTGTGATGAGCACGGCATTGACCCAACAGGTCGTTACAC
CGGCTCTTCAGACCTCCAGCTCGAGCGTGTCAATGTATACTACAATGAGGCCTCATGTGGCCGCTTTGTGCCACGTGCTGTCCTCATGGACCTTGAGCCTGGCACCATGG
ACAGTGTACGTACTGGGCCTTATGGTCAGATCTTCAGGCCTGACAACTTTGTGTTTGGGCAGTCTGGTGCTGGAAACAATTGGGCCAAGGGACACTACACTGAAGGAGCT
GAGCTGATTGATTCAGTTCTAGATGTCGT**CAGGAAGGAGGCTGAAAAT**TGTGACTGTTTGCAAGGTTTTCAGGTCTGCCATTCACTTGGTGGCGGTACTGGGTCGGGGAT
GGGAACCCTCCTGATCTCTAAGATCAGAGAGGAGTACCCTGACCGCATGATGCTG**ACCTTCTCTGTCTTCCCCTC**CCCAAAAGTTTCTGACACTGTAGTTGAGCCTTACA
ATGCAACTCTCTCTGTGCACCAGTTGGTTGAGAATGCTGATGAGTGCATGGTTCTTGATAACGAGGCACTCTATGATATTTGCTTCCGTACTCTTAAGCTCACTACACCC
AGCTTTGGAGACCTGAACCATCTGATCTCAGCCACCATGAGTGGTGTGACCTGCTGCCTGCGGTTCCCTGGTCAACTTAACTCTGACCTGCGCAAGCTTGCTGTCAACCT
TATCCCGTTCCCTCGCCTCCACTTCTTTATGGTTGGTTTTGCCCCACTTACCTCCCGTGGCTCCCAGCAGTACCGTGCACTTACTGTTCCTGAACTCACCCAACAAATGT
GGGATTCTAAGAACATGATGTGTGCGGCTGATCCCCGCCACGGTCGTTACCTTACCGCCTCTGCCATGTTCCGAGGCAAAATGAGCACAAAGGAGGTTGATGAGCAGATG
ATCAATGTGCAGAACAAGAACTCATCATACTTTGTTGAGTGGATTCCCAACAATGTGAAGTCAAGTGTGTGTGACATCCCACCAAGGGGTCTGTCAATGGCTTCTACATT
CATTGGTAACTCAACATCCATCCAGGAGATGTTCAGGAGGGTGAGTGAGCAGTTCACTGCCATGTTCCGGCGCAAGGCCTTCTTGCACTGGTACACTGGTGAGGGCATGG
ATGAGATGGAGTTCACTGAGGCTGAGAGCAACATGAATGATCTTGTGTCAGAGTACCAGCAGTACCAGGATGCAACTGCTGATGAAGAGGGTGAGTATGAGGAAGAGGAA
CTGGAGCAGGACATGTGA

UCE2 (ubiquitin-conjugating enzyme 2)

CGATCCATCGACGACTCTTCTCTTTTCTCCTCCTCTCCTCTTTCTCTCTCGCTCGAAGGCAAGAAATCGACGAAAACCAATCAATCAATCAAGAAAGGAACAAGGGACCA
ACCAACCCTCGATCGATCGATCGATCCATCGGCGGCCGATCGGATGGCGAACAGCAACCTCCCCAGACGG**ATCATCAAGGAGACCCAGC**GGCTGCTCAGCGAACCCGCTC
CTGGTATAAGTGCATCTCCTTCTGAAGATAACATGCGCTATTTTAATGTTATGATATTGGGGCCTGCACAGTCACCTTATGAAGGAGGGGTTTTCAAGCTAGAACTGTTC
TTGCCCGAAGAAT**ATCCTATGGCTGCCCCTAAG**GTTAGGTTCCTGACGAAGATTTACCATCCCAACATTGACAAGTTGGGAAGAATTTGCCTTGATATCTTGAAGGATAA
GTGGAGCCCTGCACTTCAGATTCGCACAGTGCTTTTGAGCATCCAGGCACTCCTGAGCGCTCCAAACCCTGATGATCCTTTGTCTGACAACATCGCCAAGCATTGGAAGT
CAAATGAAGCAGAAGCTGTAGAAACTGCAAAAGAGTGGACCCGCCTGTATGCAACCGGTGCATAA

UBL5 (ubiquitin-like protein 5)

TGATAAAAGATCATTATAAAGACGTACCCCGGTAAGTTTTCACTGAGCACCAATGCATCAAGAGAATCCTAAATTACCAGTATGCAAAGCACACAAACATAAAAAAGAGA
AACAAAAGCATCCAGAAAAGACATCACAACAATTGTATTTGATTATACTACCAGCACAACAGTGCAATCATATACATGCAAACACACCAAAGGTTTTGGCAATTAGAGTT
TACTTGAAGACAAGCAATCAAGAGTTATTAATAGTCACTTCACTGTGCCACACACAACTAT**ATAATCCCCGTATTTCCACTGC**AATCCTTCATAATCAAGCATTTCTTCG
TGATGAATCGGAAAGAGAAGAGA**AGAGAGCGTGGATAGGATAGATTC**AGTTGTAGTAGAGCTCGAGGCCCATGCCGTCGTGGATCTCGTAGTCCTTGAGCGTGATGTGGT
CCTTGTAAATGTTGTACCACTTCTGGATGCGGATCTTCTCGGGGCGGGTGCCGGTCTGCGCCGCCACGAGCTTCTTGAGGTCGCCGATGGTGTCGTCCTCGTTGCACTTG
ACGCGCACCTTCTTCCCCAGCCGGTCGTTCAGCACCACCTCGATCATCTTGAACGGCGATTGCGAAGTGGAGGAGTCGGGAGCTGCGGTTTGTGGCTTGGAGCGAGCCGT
GGCGGTGAGTGGATTCGATTCTAGGGTTAGGG

Table S2 Cycle threshold (Ct) values for 14 candidate reference genes.

YN tissues	RGs and Ct values													
	18S	*ACT*	*ADF7*	*CYC*	*EF1α*	*EF2*	*GAPDH*	*MDH*	*PGK*	*RPL11*	*Rubisco*	*TUB4*	*UCE2*	*UBL5*
YL	8.12 ± 0.14	15.08 ± 0.13	20.88 ± 0.09	25.34 ± 0.15	17.47 ± 0.04	18.02 ± 0.14	17.99 ± 0.12	22.91 ± 0.14	17.31 ± 0.12	18.88 ± 0.19	13.81 ± 0.13	18.18 ± 0.12	21.99 ± 0.13	17.91 ± 0.11
ML	8.49 ± 0.04	14.09 ± 0.05	20.96 ± 0.12	25.62 ± 0.03	16.24 ± 0.14	17.08 ± 0.13	17.07 ± 0.18	22.26 ± 0.19	17.84 ± 0.15	19.26 ± 0.10	14.47 ± 0.10	20.33 ± 0.07	22.21 ± 0.16	17.68 ± 0.03
SH	8.97 ± 0.15	15.94 ± 0.03	21.30 ± 0.07	25.32 ± 0.16	17.99 ± 0.18	18.62 ± 0.18	19.74 ± 0.15	23.43 ± 0.11	19.60 ± 0.11	20.06 ± 0.04	15.45 ± 0.08	19.30 ± 0.05	22.82 ± 0.05	18.50 ± 0.07
SA	7.09 ± 0.04	16.00 ± 0.07	21.88 ± 0.15	24.88 ± 0.15	19.2 ± 0.14	17.06 ± 0.11	18.17 ± 0.10	22.88 ± 0.06	23.31 ± 0.14	18.41 ± 0.19	19.75 ± 0.07	21.12 ± 0.12	22.34 ± 0.13	19.23 ± 0.11
T-form	8.30 ± 0.13	14.38 ± 0.14	21.23 ± 0.15	25.12 ± 0.20	20.00 ± 0.09	17.90 ± 0.17	18.02 ± 0.14	22.66 ± 0.12	22.61 ± 0.13	18.06 ± 0.16	16.45 ± 0.08	20.26 ± 0.13	22.51 ± 0.13	19.56 ± 0.20
T-swell	8.42 ± 0.08	15.88 ± 0.06	21.22 ± 0.03	24.98 ± 0.04	19.11 ± 0.07	17.62 ± 0.18	17.00 ± 0.06	22.00 ± 0.03	22.74 ± 0.06	17.94 ± 0.14	17.31 ± 0.16	20.73 ± 0.06	22.31 ± 0.03	19.08 ± 0.06
T-mature	8.18 ± 0.05	16.97 ± 0.18	22.10 ± 0.13	26.62 ± 0.04	21.34 ± 0.20	18.29 ± 0.20	18.39 ± 0.13	22.97 ± 0.04	23.84 ± 0.08	19.72 ± 0.07	18.39 ± 0.15	22.41 ± 0.04	23.50 ± 0.06	20.75 ± 0.14
RH	8.59 ± 0.03	15.60 ± 0.16	21.27 ± 0.15	24.45 ± 0.06	18.47 ± 0.19	17.18 ± 0.08	18.32 ± 0.16	21.80 ± 0.05	22.40 ± 0.09	17.97 ± 0.18	19.41 ± 0.16	19.78 ± 0.13	22.06 ± 0.12	19.12 ± 0.18
R	9.45 ± 0.12	16.47 ± 0.03	22.31 ± 0.03	24.37 ± 0.13	19.34 ± 0.14	18.28 ± 0.11	18.58 ± 0.14	22.20 ± 0.04	23.85 ± 0.06	18.33 ± 0.15	20.95 ± 0.06	22.19 ± 0.20	22.61 ± 0.12	19.21 ± 0.04
Average	8.40 ± 0.61	15.60 ± 0.88	21.46 ± 0.48	25.19 ± 0.64	18.80 ± 1.40	17.78 ± 0.55	18.14 ± 0.77	22.57 ± 0.50	21.50 ± 2.41	18.74 ± 0.74	17.33 ± 2.34	20.48 ± 1.26	22.48 ± 0.44	19.00 ± 0.86
**XJ tissues**	**RGs and Ct values**													
	18S	*ACT*	*ADF7*	*CYC*	*EF1α*	*EF2*	*GAPDH*	*MDH*	*PGK*	*RPL11*	*Rubisco*	*TUB4*	*UCE2*	*UBL5*
YY	9.11 ± 0.09	20.72 ± 0.12	20.06 ± 0.15	26.29 ± 0.27	20.06 ± 0.24	19.47 ± 0.19	20.73 ± 0.16	24.63 ± 0.19	18.53 ± 0.07	22.03 ± 0.25	15.12 ± 0.27	21.02 ± 0.07	24.27 ± 0.06	19.02 ± 0.16
MY	9.16 ± 0.25	22.17 ± 0.11	19.89 ± 0.13	26.91 ± 0.06	19.16 ± 0.12	20.19 ± 0.26	21.46 ± 0.12	24.46 ± 0.17	18.38 ± 0.21	23.12 ± 0.16	15.32 ± 0.25	23.46 ± 0.25	24.37 ± 0.18	18.81 ± 0.27
SH	9.29 ± 0.23	23.76 ± 0.17	19.07 ± 0.11	26.53 ± 0.09	20.27 ± 0.14	19.61 ± 0.17	21.35 ± 0.25	24.06 ± 0.24	19.32 ± 0.21	21.00 ± 0.11	17.05 ± 0.07	17.04 ± 0.06	24.76 ± 0.09	19.87 ± 0.06
SA	9.31 ± 0.06	22.47 ± 0.12	20.01 ± 0.09	25.91 ± 0.19	20.55 ± 0.07	19.71 ± 0.22	20.65 ± 0.12	24.14 ± 0.16	20.46 ± 0.17	21.99 ± 0.11	20.97 ± 0.26	21.71 ± 0.18	25.05 ± 0.24	20.02 ± 0.11
T-form	8.44 ± 0.21	19.32 ± 0.11	18.45 ± 0.21	24.65 ± 0.25	20.55 ± 0.23	18.70 ± 0.19	19.96 ± 0.25	24.37 ± 0.19	20.88 ± 0.24	20.08 ± 0.12	15.84 ± 0.25	20.95 ± 0.06	23.21 ± 0.26	18.42 ± 0.07
T-swell	8.96 ± 0.14	18.04 ± 0.18	18.36 ± 0.27	25.22 ± 0.08	19.95 ± 0.27	18.92 ± 0.23	19.71 ± 0.09	25.02 ± 0.25	21.28 ± 0.14	19.37 ± 0.26	15.36 ± 0.22	20.63 ± 0.06	23.83 ± 0.17	18.26 ± 0.15
T-mature	10.55 ± 0.14	18.45 ± 0.09	20.28 ± 0.17	26.01 ± 0.26	22.33 ± 0.18	21.37 ± 0.07	20.26 ± 0.09	25.72 ± 0.15	22.10 ± 0.08	20.88 ± 0.15	16.92 ± 0.09	21.71 ± 0.22	23.83 ± 0.22	20.03 ± 0.15
RH	10.54 ± 0.06	20.77 ± 0.25	20.34 ± 0.12	26.82 ± 0.16	21.10 ± 0.17	20.99 ± 0.13	21.79 ± 0.06	24.02 ± 0.27	22.89 ± 0.15	22.05 ± 0.21	22.98 ± 0.24	21.96 ± 0.09	24.56 ± 0.12	19.22 ± 0.19
R	9.79 ± 0.27	19.75 ± 0.15	19.92 ± 0.24	27.87 ± 0.24	19.78 ± 0.24	19.49 ± 0.19	22.98 ± 0.19	25.36 ± 0.24	19.74 ± 0.14	19.50 ± 0.18	18.19 ± 0.13	19.59 ± 0.07	23.00 ± 0.19	20.10 ± 0.25
Average	9.46 ± 0.67	20.61 ± 1.81	19.60 ± 0.72	26.24 ± 0.91	20.42 ± 0.85	19.83 ± 0.83	20.99 ± 0.97	24.64 ± 0.57	20.40 ± 1.46	21.11 ± 1.22	17.53 ± 2.60	20.90 ± 1.69	24.10 ± 0.65	19.30 ± 0.68

YL: young leaves, ML: mature leaves, SH: sheath, SA: shoot apexes, T-form: tubers in formation stage, T-swell: tubers in swelling stage, T-mature: tubers in mature stage, RH: rhizome, R: root

Table S3 Ranking orders of 14 reference genes were integrated by geNorm, NormFinder, BestKeeper and RankAggreg in YN tiger nut.

Analysis Tool	Ranking Order of YN (the 1st is the most stable, and the 12th is the least stable)													
	**1**	**2**	**3**	**4**	**5**	**6**	**7**	**8**	**9**	**10**	**11**	**12**	**13**	**14**
**Overground***														
geNorm	*UCE2*	*MDH*	*UBL5*	*ADF7*	*ACT*	*CYC*	*TUB4*	*EF2*	*EF1α*	*RPL11*	*GAPDH*	*18S*	*PGK*	*Rubisco*
NormFinder	*UCE2*	*UBL5*	*MDH*	*ADF7*	*EF1α*	*ACT*	*GAPDH*	*CYC*	*EF2*	*TUB4*	*RPL11*	*18S*	*PGK*	*Rubisco*
BestKeeper	*MDH*	*CYC*	*UCE2*	*ADF7*	*UBL5*	*EF2*	*18s*	*RPL11*	*ACT*	*GAPDH*	*EF1α*	*TUB4*	*Rubisco*	*PGK*
RankAggreg	*UCE2*	*MDH*	*UBL5*	*ADF7*	*ACT*	*EF2*	*CYC*	*EF1α*	*TUB4*	*GAPDH*	*RPL11*	*18S*	*PGK*	*Rubisco*
**Underground**														
geNorm	*UCE2*	*TUB4*	*ACT*	*EF2*	*MDH*	*RPL11*	*CYC*	*EF1α*	*GAPDH*	*UBL5*	*18S*	*PGK*	*ADF7*	*Rubisco*
NormFinder	*RPL11*	*TUB4*	*MDH*	*UCE2*	*ACT*	*EF2*	*GAPDH*	*CYC*	*EF1α*	*UBL5*	*PGK*	*18S*	*ADF7*	*Rubisco*
BestKeeper	*UCE2*	*MDH*	*ACT*	*TUB4*	*ADF7*	*EF2*	*GAPDH*	*PGK*	*UBL5*	*RPL11*	*CYC*	*18S*	*EF1α*	*Rubisco*
RankAggreg	*UCE2*	*TUB4*	*MDH*	*ACT*	*EF2*	*RPL11*	*GAPDH*	*CYC*	*UBL5*	*EF1α*	*PGK*	*18S*	*ADF7*	*Rubisco*
**Tuber**														
geNorm	*UCE2*	*TUB4*	*ACT*	*EF2*	*MDH*	*GAPDH*	*CYC*	*RPL11*	*Rubisco*	*EF1α*	*UBL5*	*18S*	*PGK*	*ADF7*
NormFinder	*RPL11*	*TUB4*	*UCE2*	*MDH*	*EF2*	*EF1α*	*GAPDH*	*ACT*	*UBL5*	*CYC*	*Rubisco*	*PGK*	*18S*	*ADF7*
BestKeeper	*TUB4*	*UCE2*	*EF2*	*MDH*	*18S*	*GAPDH*	*CYC*	*RPL11*	*Rubisco*	*EF1α*	*UBL5*	*ACT*	*PGK*	*ADF7*
RankAggreg	*TUB4*	*UCE2*	*EF2*	*MDH*	*RPL11*	*GAPDH*	*ACT*	*CYC*	*EF1α*	*Rubisco*	*UBL5*	*18S*	*PGK*	*ADF7*
**Total**														
geNorm	*UCE2*	*MDH*	*TUB4*	*UBL5*	*CYC*	*EF2*	*RPL11*	*GAPDH*	*18S*	*ACT*	*ADF7*	*EF1α*	*PGK*	*Rubisco*
NormFinder	*UCE2*	*UBL5*	*EF2*	*MDH*	*ACT*	*ADF7*	*GAPDH*	*CYC*	*EF1α*	*TUB4*	*RPL11*	*18S*	*PGK*	*Rubisco*
BestKeeper	*UCE2*	*MDH*	*EF2*	*18S*	*UBL5*	*CYC*	*RPL11*	*GAPDH*	*ADF7*	*ACT*	*TUB4*	*EF1α*	*Rubisco*	*PGK*
RankAggreg	*UCE2*	*MDH*	*UBL5*	*EF2*	*CYC*	*GAPDH*	*TUB4*	*ACT*	*RPL11*	*18S*	*ADF7*	*EF1α*	*PGK*	*Rubisco*

* Overground part including young leaf, mature leaf, leaf sheath and stem apex. Underground part including root, rhizome and all tubers. Tuber part contains three developmental stages of tubers collected at 40, 80 and 120 days after sowing (DAS), respec

Table S4 Ranking orders of 14 reference genes was integrated by geNorm, NormFinder, BestKeeper and RankAggreg in XJ tiger nut.

Analysis Tool	Ranking Order of XJ (the 1st is the most stable, and the 12th is the least stable)													
	**1**	**2**	**3**	**4**	**5**	**6**	**7**	**8**	**9**	**10**	**11**	**12**	**13**	**14**
	**Overground***													
geNorm	*UCE2*	*UBL5*	*EF1α*	*CYC*	*GAPDH*	*ACT*	*MDH*	*ADF7*	*TUB4*	*18S*	*EF2*	*RPL11*	*PGK*	*Rubisco*
NormFinder	*UCE2*	*EF1α*	*CYC*	*PGK*	*ACT*	*MDH*	*RPL11*	*TUB4*	*EF2*	*GAPDH*	*UBL5*	*18S*	*Rubisco*	*ADF7*
BestKeeper	*MDH*	*EF2*	*UCE2*	*GAPDH*	*CYC*	*18S*	*ADF7*	*EF1α*	*UBL5*	*RPL11*	*TUB4*	*PGK*	*ACT*	*Rubisco*
RankAggreg	*UCE2*	*EF1α*	*CYC*	*MDH*	*EF2*	*UBL5*	*GAPDH*	*ACT*	*18S*	*PGK*	*RPL11*	*ADF7*	*TUB4*	*Rubisco*
	**Underground**													
geNorm	*UCE2*	*TUB4*	*ACT*	*UBL5*	*ADF7*	*18S*	*EF1α*	*PGK*	*RPL11*	*CYC*	*EF2*	*MDH*	*GAPDH*	*Rubisco*
NormFinder	*ADF7*	*TUB4*	*UBL5*	*UCE2*	*CYC*	*EF1α*	*RPL11*	*18S*	*ACT*	*EF2*	*MDH*	*PGK*	*GAPDH*	*Rubisco*
BestKeeper	*UCE2*	*MDH*	*UBL5*	*TUB4*	*18S*	*ADF7*	*EF1α*	*ACT*	*RPL11*	*PGK*	*EF2*	*CYC*	*GAPDH*	*Rubisco*
RankAggreg	*UCE2*	*UBL5*	*TUB4*	*ADF7*	*18S*	*ACT*	*EF1α*	*RPL11*	*MDH*	*CYC*	*PGK*	*EF2*	*GAPDH*	*Rubisco*
	**Tuber**													
geNorm	*TUB4*	*UBL5*	*UCE2*	*PGK*	*ADF7*	*CYC*	*RPL11*	*MDH*	*EF1α*	*EF2*	*ACT*	*18S*	*GAPDH*	*Rubisco*
NormFinder	*TUB4*	*PGK*	*ADF7*	*CYC*	*UBL5*	*UCE2*	*RPL11*	*MDH*	*EF1α*	*ACT*	*EF2*	*18S*	*GAPDH*	*Rubisco*
BestKeeper	*UCE2*	*TUB4*	*PGK*	*ACT*	*MDH*	*CYC*	*RPL11*	*GAPDH*	*UBL5*	*ADF7*	*18S*	*EF1α*	*EF2*	*Rubisco*
RankAggreg	*TUB4*	*PGK*	*UCE2*	*CYC*	*UBL5*	*ADF7*	*MDH*	*ACT*	*RPL11*	*EF1α*	*EF2*	*GAPDH*	*18S*	*Rubisco*
	**Total**													
geNorm	*UCE2*	*ADF7*	*UBL5*	*18S*	*CYC*	*EF2*	*GAPDH*	*TUB4*	*EF1α*	*MDH*	*RPL11*	*PGK*	*ACT*	*Rubisco*
NormFinder	*UBL5*	*UCE2*	*ADF7*	*CYC*	*EF2*	*18S*	*ACT*	*GAPDH*	*EF1α*	*TUB4*	*MDH*	*RPL11*	*Rubisco*	*PGK*
BestKeeper	*UCE2*	*18S*	*UBL5*	*ADF7*	*EF2*	*EF1α*	*MDH*	*CYC*	*GAPDH*	*RPL11*	*PGK*	*TUB4*	*ACT*	*Rubisco*
RankAggreg	*UCE2*	*UBL5*	*ADF7*	*18S*	*EF2*	*CYC*	*EF1α*	*GAPDH*	*MDH*	*TUB4*	*ACT*	*RPL11*	*PGK*	*Rubisco*

* Overground part including young leaf, mature leaf, leaf sheath and stem apex. Underground part including root, rhizome and all tubers. Tuber part contains three developmental stages of tubers collected at 40, 80 and 120 days after sowing (DAS).

Table S5 The concentrations and the volumes of total RNA for cDNA synthesis

Sample of XJ	Total RNA concentrations (ng/μL)	Total RNA volumes for the cDNA production （μL）	RNase Free H_2O （μL）	Sample of YN	Total RNA concentrations (ng/μL)	Total RNA volumes for the cDNA production （μL）	RNase Free H_2O （μL）
YL-1	413.7	5	9	YL-1	1223.4	1.6	12.4
YL-2	1178.4	1.8	12.2	YL-2	1426.6	1.4	12.6
YL-3	1727.6	1.2	12.8	YL-3	1239	1.6	12.4
ML-1	1015.4	2	12	ML-1	605.1	3.2	10.8
ML-2	1109.8	2	12	ML-2	1213.5	1.6	12.4
ML-3	917.8	2.2	11.8	ML-3	998.6	2	12
SH-1	1405.6	1.4	12.6	SH-1	1012.3	2	12
SH-2	1737.8	1.2	12.8	SH-2	455.7	4.4	9.6
SH-3	1962.5	1.1	12.9	SH-3	468.2	4.4	9.6
SA-1	1580.1	1.4	12.6	SA-1	913.5	1.2	12.8
SA-2	1644.4	1.2	12.8	SA-2	1182.3	1.8	12.2
SA-3	1868.9	1.1	12.9	SA-3	1229.6	1.6	12.4
T-form-1	491.7	4.1	9.9	T-form-1	716.8	2.8	11.2
T-form-2	622	3.3	10.7	T-form-2	1125.8	1.8	12.2
T-form-3	698.3	2.9	11.1	T-form-3	1234.6	1.6	12.4
T-swell-1	1817.3	1.1	12.9	T-swell-1	1240.2	1.8	12.2
T-swell-2	1678.2	1.2	12.8	T-swell-2	829.8	2.4	11.6
T-swell-3	1575.9	1.2	12.8	T-swell-3	841.9	2.4	11.6
T-mature-1	1168.4	1.7	12.3	T-mature-1	1257.9	1.6	12.4
T-mature-2	1021.3	2	12	T-mature-2	1244.1	1.6	12.4
T-mature-3	498.2	4	10	T-mature-3	1120.8	1.8	12.2
RH-1	735.5	2.7	11.3	RH-1	622.3	3.2	10.8
RH-2	339.2	6	8	RH-2	627.8	3.2	10.8

RH-3	345.8	5.8	8.2	RH-3	476.3	4.2	9.8
R-1	437.7	4.7	9.3	R-1	434.8	4.6	9.4
R-2	418.2	5	9	R-2	588.3	3.4	10.6
R-3	392.5	5	9	R-3	523.9	3.8	10.2
